# Role of p38 MAPK in enhanced human cancer cells killing by the combination of aspirin and ABT-737

**DOI:** 10.1111/jcmm.12461

**Published:** 2014-11-11

**Authors:** Chong Zhang, Jing Shi, Shi-ying Mao, Ya-si Xu, Dan Zhang, Lin-yi Feng, Bo Zhang, You-you Yan, Si-cong Wang, Jian-ping Pan, You-ping Yang, Neng-ming Lin

**Affiliations:** a School of Medicine, Zhejiang University City CollegeHangzhou, Zhejiang, China; b Department of Pharmacy, Zhejiang Medical CollegeHangzhou, Zhejiang, China; c Institute for Individualized Medicine, Hangzhou First People's HospitalHangzhou, Zhejiang, China; d Affiliated Hangzhou First People's Hospital of Zhejiang Chinese Medical UniversityHangzhou, Zhejiang, China; e Laboratory of Clinical Pharmacy, Zhejiang Cancer HospitalHangzhou, Zhejiang, China

**Keywords:** aspirin, ABT-737, combination, p38

## Abstract

Regular use of aspirin after diagnosis is associated with longer survival among patients with mutated-PIK3CA colorectal cancer, but not among patients with wild-type PIK3CA cancer. In this study, we showed that clinically achievable concentrations of aspirin and ABT-737 in combination could induce a synergistic growth arrest in several human PIK3CA wild-type cancer cells. In addition, our results also demonstrated that long-term combination treatment with aspirin and ABT-737 could synergistically induce apoptosis both in A549 and H1299 cells. In the meanwhile, short-term aspirin plus ABT-737 combination treatment induced a greater autophagic response than did either drug alone and the combination-induced autophagy switched from a cytoprotective signal to a death-promoting signal. Furthermore, we showed that p38 acted as a switch between two different types of cell death (autophagy and apoptosis) induced by aspirin plus ABT-737. Moreover, the increased anti-cancer efficacy of aspirin combined with ABT-737 was further validated in a human lung cancer A549 xenograft model. We hope that this synergy may contribute to failure of aspirin cancer therapy and ultimately lead to efficacious regimens for cancer therapy.

## Introduction

Aspirin, a non-selective COX inhibitor, has been successfully used as an anti-inflammatory drug for more than 100 years 1. Recently, aspirin and some other non-steroidal anti-inflammatory drugs (NSAIDs) have drawn much attention for their anti-tumour effects 2. Aspirin therapy has been associated with a superior clinical outcome by reducing the occurrence of several malignant tumours 3–5. However, regular use of aspirin after diagnosis is associated with longer survival among patients with mutated-PIK3CA colorectal cancer, but not among patients with wild-type PIK3CA cancer 6. Recent reports also reveal that combination of aspirin with conventional chemotherapeutic agents, such as sorafenib and TRAIL, can exhibit enhanced anti-cancer effects both in PIK3CA mutant and PIK3CA wild-type cancer cell lines 2,7. Thus, combining aspirin with a chemotherapeutic agent may be a logical way to potentially enhance response rates and prolong survival times for patients with cancer.

Autophagy and apoptosis have been shown to be coincident or antagonistic, depending on experimental context, and share cross-talk between signal transduction elements 8. Many molecules, such as AKT and p38, can activate pathways that affect both apoptosis and autophagy 9. P38 MAPK, one of three distinct families of MAPKs (ERK, JNK and p38 kinase), plays an important role in cell cycle arrest, apoptosis and autophagy in response to different stimuli 10,11. Furthermore, p38 MAP kinase plays a vital role in the switch from autophagy to apoptosis in cancer cells response to anti-cancer drug treatment 12.

The BH3-mimetic ABT-737 and an orally bioavailable compound of the same class, navitoclax (ABT-263), are small-molecule chemicals that mimic the direct binding to the hydrophobic groove in Bcl-2, Bcl-xL and Bcl-w. ABT-737 can induce apoptosis in multiple cancer cells 13. Recent finding has revealed that aspirin could enhance ABT-263-mediated anti-cancer activity *via* suppression of Mcl-1 in hepatocellular carcinoma 14. In this context, we investigated whether aspirin + ABT-737 could synergistically inhibit the proliferation in other cancer cells with different genetic backgrounds. Besides, our data showed that inhibition of Mcl-1 by aspirin + ABT-737 might differ depending on the cell type. We also demonstrated that long-term combination treatment with aspirin and ABT-737 induced apoptosis through mitochondrial pathway and short-term combination treatment induced autophagy both in A549 and H1299 cells. In addition, p38 kinase might act as a switch in the transition between autophagy and apoptosis in A549 cells treated with aspirin + ABT-737. We hope that this synergy may ultimately lead to efficacious regimens for cancer therapy.

## Materials and methods

### Materials

Aspirin from Sigma-Aldrich (St. Louis, MO, USA) was dissolved in DMSO and the pH was adjusted to 7.0 using 10 N NaOH. ABT-737 was synthesized according to the literature and its purity was greater than 99% as assessed by HPLC 15. 3-Methyladenine (3-MA) and 4′-6-Diamidino-2-phenylindole (DAPI) were purchased from Sigma-Aldrich. Baflomycine A1 was purchased from BioVision (Milpitas, CA, USA). The p38 MAPK inhibitor (SB-203580) was purchased from Selleck Chemicals (Houston, TX, USA). The primary antibodies against p38, Mcl-1, PARP, procaspase-3, XIAP and HRP-labelled secondary antimouse and anti-rabbit antibodies were purchased from Santa Cruz Biotechnology (Dallas, TX, USA); p-p38(Thr-180/Tyr-182), LC-3, cytochrome C and cleaved caspase-3 from Cell Signaling Technology (Danvers, MA, USA); and β-actin from BD Biosciences (Franklin Lakes, NJ, USA).

### Cell Culture

Human ovarian cancer cell line (HO-8910), human lung cancer cell lines (A549, H1299), human colon cancer cell lines (HCT-116, HT-29) and human normal liver cell line (Chang liver) were purchased from Shanghai institute of biochemistry and cell biology (Shanghai, China); they were tested and authenticated for genotypes by DNA fingerprinting. HO-8910, H1299, HT-29 and HCT-116 were maintained in DMEM supplemented with 10% foetal bovine serum, A549 was grown in Ham's F12 medium supplemented with 10% foetal bovine serum. All the cells were maintained in a humidified atmosphere of 95% air plus 5% CO_2_ at 37°C.

### Cytotoxicity assay

The anti-proliferative activity of combination treatment with aspirin and ABT-737 was measured by sulforhodamine blue (SRB) cytotoxicity assay. Briefly, cells were fixed with 10% TCA solution for 1 hr, wells were rinsed five times with tap water and then cells were stained with 0.4% SRB solution (100 μl per well) for 20 min. at room temperature; wells were rinsed with 1% acetic acid to remove unbound dye, and were then left to air dry; the SRB dye was then solubilized by placing 100 μl of unbuffered Tris-based solution in each well, and the absorbance was measured at 515 nm using a multi-scan spectrum. The inhibition rate of cell proliferation was calculated for each well as (A515 control cells – A515 treated cells)/A515 control cells × 100%.

### Colony formation assay

Cells were plated at 500–1000 cells/dish. The medium was replaced every 3 days at the indicated concentrations. Dishes were stained by crystal violet after 14 days treatment and colony numbers were counted.

### Analysis of apoptosis by propidium iodide staining

Cells (3 × 10^5^/well) were seeded into six-well plates and exposed to aspirin, ABT-737 or the combination. Cells were harvested and washed with PBS, fixed with pre-cooled 70% ethanol at 4°C overnight. Fixed cells were then washed with PBS to remove residual ethanol, pelleted, resuspended in 500 μl PBS containing 50 μg RNase A at 37°C and 5 μg PI in dark at room temperature for 30 min. For each sample, 2 × 10^4^ cells were collected and analysed using an FACS-Calibur cytometer (Becton Dickinson, San Jose, CA, USA).

### Determination of mitochondrial membrane depolarization

Cells (3 × 10^5^/well) were exposed to aspirin, ABT-737 or the combination for 48 hrs, collected, and resuspended in fresh medium containing 10 μg/ml 5,5′,6,6′tetrachloro-1,1′,3,3′-tetraethylbenzimidazol-carbocyanine iodide (JC-1). After incubation at 37°C for 30 min., cells were analysed by flow cytometry.

### Cell lysates and Western blot analysis

Proteins were extracted with lysis buffer (50 mM Tris–HCl, 150 mM NaCl, 1 mM EDTA, 0.1% SDS, 0.5% deoxycholic acid, 0.02% sodium azide, 1% NP-40, 2.0 μg/ml aprotinin, 1 mM phenylmethylsulfonylfluoride). The lysates were centrifuged at 10,000 × g for 30 min. at 4°C, the supernatants were transferred to a new tube and the protein concentrations were determined. To analyse cytochrome c release from mitochondria, mitochondria were extracted using Mitochondria/cytosol fractionation kit (Beyotime Institute of Biotechnology, Shang-hai, China). Proteins were fractionated on 8–15% Tris-glycine gels, and then they were transferred to PVDF membrane (Millipore, Bedford, MA, USA) and probed with primary antibodies (dilution range 1:500–1:1000) followed by horseradish peroxidase-labelled secondary antibodies at 1:5000 dilution. Antibody binding was then detected with the use of a chemiluminescent substrate and visualized on autoradiography film.

### Analysis of apoptosis by DAPI staining

Briefly, cells were washed twice with PBS and then incubated with 0.1% Triton and 0.1% DAPI. The morphology of the cells' nuclei was observed and captured using a fluorescence microscope at excitation wavelength 350 nm.

### Plasmid transfection

GFP-LC-3 plasmid was transfected into cells using Lipofectamine 2000 as recommended by the manufacturer.

### P38 gene silencing by short interfering RNA

Cells (5 × 10^4^) were incubated overnight in six-well plates. On day 2, the medium was replaced with Opti-MEM I Reduced Serum Media (GIBCO, Grand Island, NY, USA) containing 20.0 nM p38 short interfering RNA (siRNA; GenePharma, Shang-hai, China) and oligofectamine reagent (Invitrogen Corporation, Grand Island, NY, USA) according to the manufacturer's recommendations. The sense sequences of the p38 siRNA were 5′-GCAUAAUGGCCGAGCUGUUTT-3′ (p38 SiRNA-1), 5′-GAACUGCGGUUACUUAAAC-3′ (p38 SiRNA-2).

### Quantification of acidic vesicular organelles with acridine orange staining by flow cytometry

To quantify the development of acidic vesicular organelles, cells were stained with acridine orange (AO, 1 μg/ml) for 15 min. and removed from the plate with trypsin–EDTA. The stained cells were then analysed by flow cytometer.

### Animals and anti-tumour activity *in vivo*

Human lung cancer A549 xenografts were established by injecting 5 × 10^6^ cells subcutaneously into nude mice. When the tumour reached a volume of 50–150 mm^3^, the mice were randomized to control and treated groups and then received vehicle [1% DMSO, 7% Cremophor/ethanol (3:1), and 92% PBS, i.p. administration], aspirin (100 mg/kg, i.g. administration) daily and ABT-737 (50 mg/kg, i.p. administration) twice per week for 29 days (*n* = 8 per group). Tumour volume (V) was calculated as V = (length × width × height)/2. The tumour volume at day n was expressed as relative tumour volume (RTV) according to the following formula: RTV = TV_n_/TV_0_, where TV_n_ was the tumour volume at day n and TV_0_ was the tumour volume at day 0.

### Statistical analyses

Two tailed Student's *t*-tests were used to determine the significance of differences between the experiment conditions. Differences were considered significant at *P* < 0.05. Combination index was well-accepted for quantifying drug synergism based on the multiple drug-effect equation of Chou–Talalay 16. For *in vitro* experiments, CI values were calculated for each concentration of aspirin, ABT-737 and the combination in cell proliferation assays using Calcusyn (Biosoft, Cambridge, UK). Different CI values were obtained when solving the equation for different effect levels, and mean CI values were chosen for presentation. A CI less than 0.9 indicated synergism; 0.1, very strong synergism; 0.1–0.3, strong synergism; 0.3–0.7, synergism; 0.7–0.85, moderate synergism; 0.85–0.9, slight synergism; 0.9–1.10, additive; and more than 1.10, antagonism.

## Results

### Cytotoxicity of the aspirin and ABT-737 combination in human cancer cell lines

**Fig 1:**
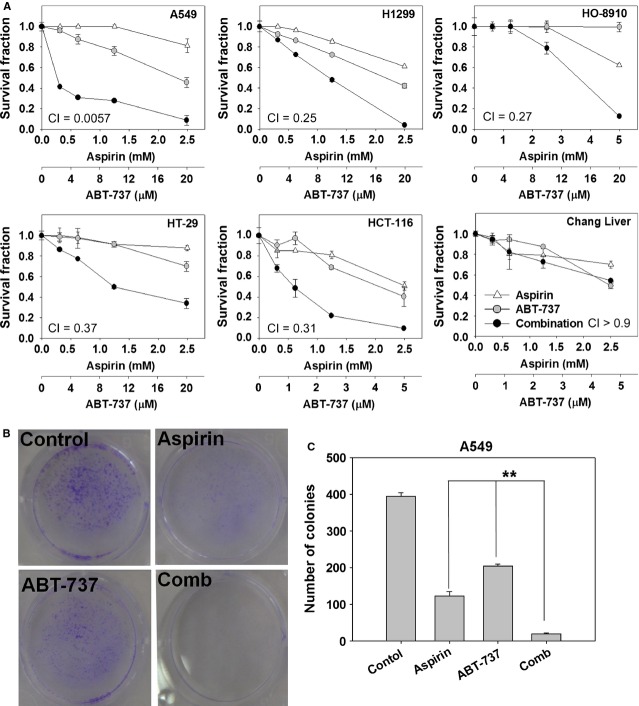
Combination cytotoxicity of aspirin and ABT-737. (A) The cells were incubated with the compounds for 72 hrs. Dose–response curves of human cancer cell lines to aspirin, ABT-737 or the combination were showed. Each condition had six replicates and error bars represented standard deviation. (B) Combination treatment with aspirin and ABT-737 inhibited the colony formation in A549 cells. Cells were treated with aspirin (1.25 mM), ABT-737 (2.5 μM) or the combination for 14 days. (C) Changes in the number of colonies formed by A549 cells after treatments. **P < 0.01, mono-treatment *versus* combination treatment.

The sensitivities of five human cancer cell lines to aspirin, ABT-737, or aspirin + ABT-737 were determined by SRB cytotoxicity assay. Survival curves to aspirin, ABT-737 and aspirin combined with ABT-737 were shown in Figure1A. CI values were calculated using Calcusyn at the fixed-ratio concentrations of aspirin and ABT-737 to assess combination activity. Aspirin plus ABT-737 showed synergistic cytotoxic effect in five human cancer cell lines, with the mean CI values below 0.7. Moreover, aspirin plus ABT-737 showed no synergy against normal Chang liver cells (CI >0.9), suggesting that aspirin plus ABT-737 might improve the anti-tumour effect without increasing toxicity. In a long-term colony formation assay, aspirin at 1.25 mM and ABT-737 at 2.5 μM alone had partial effects on suppression of colony formation of A549 cells; however, the combination almost eliminated colony formation (Fig.1B and C). Collectively, these data supported the combination was much more effective in inhibiting the proliferation of cancer cells than either single agent.

### Long-term combination treatment with aspirin and ABT-737 induced apoptosis through mitochondrial pathway both in A549 and H1299 cells

**Fig 2:**
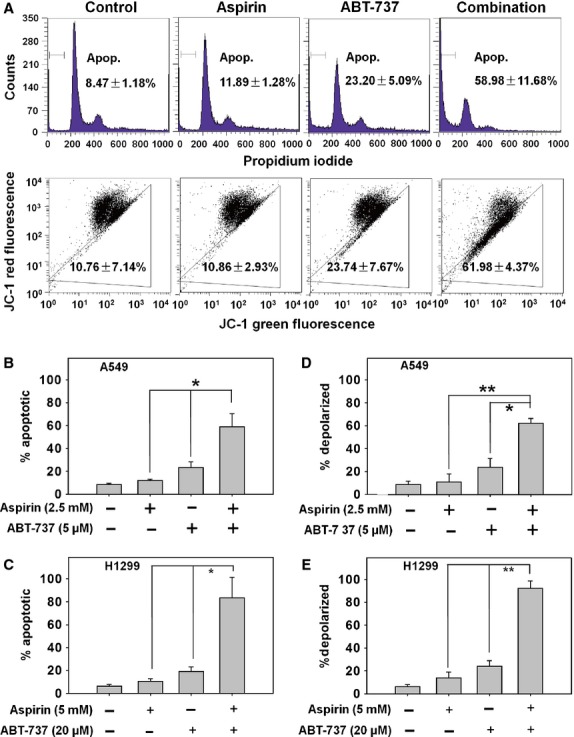
Long-term combination treatment with aspirin and ABT-737 caused enhanced apoptosis. (A) A549 cells were treated with aspirin (2.5 mM), ABT-737 (5 μM) or the combination for 48 hrs. Cells were stained with PI (upper) or JC-1 (bottom) and analysed by flow cytometry. A549 (B) and H1299 (C) cells in six-well plates were exposed to the compounds for 48 hrs and then cells were analysed by flow cytometry after PI staining. A549 (D) and H1299 (E) cells in six-well plates were exposed to compounds for 48 hrs and then cells were analysed by flow cytometry after JC-1 staining. The experiments were repeated three times and error bars represented standard deviation. *P < 0.05, **P < 0.01.

**Fig 3:**
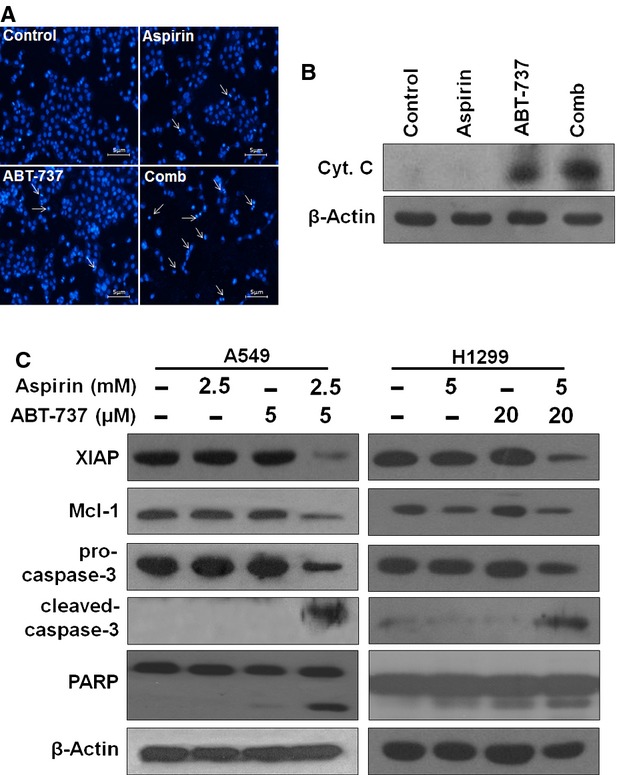
Long-term combination treatment with aspirin and ABT-737 caused activation of various apoptosis related proteins. (A) Aspirin plus ABT-737 induced apoptotic bodies in A549 cells. Nuclear DNA was visualized by DAPI staining; scale bar = 5 μm. (B) Cytochrome c release from mitochondria into cytosol was assessed at 48 hrs in A549 cells incubated with the agents. Cytosolic protein extracts were immunoblotted with specified antibodies for cytochrome c and β-actin. (C) A549 and H1299 cells were exposed to aspirin, ABT-737 or the combination for 48 hrs, after which protein extracts were immunoblotted with specified antibodies for XIAP, Mcl-1, caspase-3, cleaved caspase-3, PARP and β-actin.

To explore the mechanism of synergistic effects by combining aspirin and ABT-737, we first detected apoptosis by PI staining in A549 and H1299 cells that displayed strong synergistic effects in the cytotoxicity assay. As shown in Figure2A (top panel), PI staining was used to characterize the apoptosis in A549 cells treated with 2.5 mM aspirin, 5 μM ABT-737 or the combination for 48 hrs, the percentage of apoptotic cells was 8.47% in control cells, 11.89% with aspirin, 23.20% with ABT-737 and 58.98% in the combination treatment group. The apoptotic A549 and H1299 cells were significantly higher in the combination treatment when compared to single treatment (Fig.2B and C, *P* < 0.05). To further explore the mechanisms of enhanced apoptosis seen with the combined treatment, we next investigated the effect of aspirin and ABT-737 on the loss of mitochondrial membrane potential (Δψm) in A549 cells. As shown in Figure2A (bottom panel) and Figure2D, combined treatment with aspirin and ABT-737 resulted in an increased percentage of mitochondrial membrane depolarized A549 cells than either agent used alone (61.98% in combination-treated cells, 10.86% in aspirin-treated cells, 23.74% in ABT-737-treated cells and 10.76% in control group). Similar results were obtained in H1299 cells (Fig.2E). In addition, DAPI staining was also performed to visualize the apoptosis by assessing chromatin condensation. As shown in Figure3A, aspirin plus ABT-737 triggered more apoptosis than the mono-treatment in A549 cells, as indicated by the apoptotic bodies. These results suggested mitochondrial apoptotic pathway might be involved in the synergistic effect by combining aspirin and ABT-737. To further confirm this, cytochrome c release from mitochondria to the cytosol in response to treatment was measured. The level of cytosolic cytochrome c was greater in A549 cells treated with aspirin + ABT-737 than cells treated with aspirin or ABT-737 as single agents (Fig.3B). Moreover, immunoblot analysis was performed to determine whether enhanced ABT-737-mediated apoptosis by aspirin involved caspase activation. Combination treatment markedly induced the activation of caspase-3 and cleavage of PARP (Fig.3C). In the meanwhile, combination therapy also resulted in apoptotic induction with XIAP inhibition both in A549 and H1299 cells. However, aspirin + ABT-737 resulted in a greater Mcl-1 reduction compared with the single agents only in A549 cells, but not in H1299 cells.

### Short-term combination treatment with aspirin and ABT-737 induced autophagy both in A549 and H1299 cells

**Fig 4:**
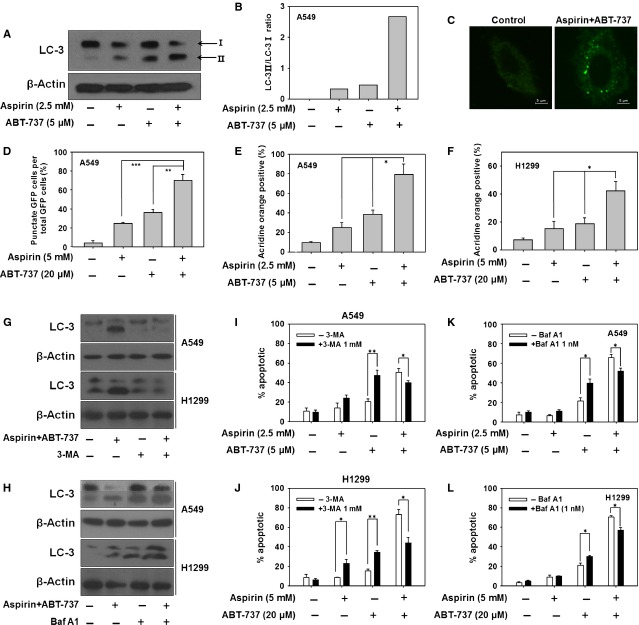
Short-term combination treatment with aspirin and ABT-737-induced autophagy. (A) A549 cells were exposed to aspirin (2.5 mM), ABT-737 (5 μM) or the combination for 12 hrs, after which protein extracts were immunoblotted using LC-3. (B) LC-3 II/I ratios were significantly increased in combination treatment with aspirin (2.5 mM) plus ABT-737 (5 μM) for 12 hrs in A549 cells. (C) A549 cells were transfected with GFP-LC-3 plasmid according to manufacturer's recommendations. Twenty-four hours after transfection, cells were exposed to aspirin (2.5 mM), ABT-737 (5 μM) or the combination for 12 hrs. Aspirin plus ABT-737 induced GFP-LC3 dot formation in A549 cells; scale bar = 5 μm. (D) Quantified results of punctate GFP-LC3/A549 cells undergoing aspirin and/or ABT-737 treatment were measured (mean ± SD, *n* = 3). ***P < 0.001, **P < 0.01. A549 (E) and H1299 (F) cells treated with compounds for 12 hrs were stained with AO and analysed by flow cytometry. (G) A549 and H1299 cells were pre-treated with 1 mM 3-MA for 1 hr and incubated with aspirin and ABT-737 at the indicated concentrations for 12 hrs, after which protein extracts were immunoblotted using LC-3. (H) A549 and H1299 cells were pre-treated with 1 nM Baflomycine A1 for 1 hr and incubated with aspirin and ABT-737 at the indicated concentrations for 12 hrs, after which protein extracts were immunoblotted using LC-3. A549 (I) and H1299 (J) cells were pre-treated with 1 mM 3-MA for 1 hr and incubated with aspirin and/or ABT-737 at the indicated concentrations for 48 hrs. Percentages of apoptotic cells were determined by PI analysis. A549 (K) and H1299 (L) cells were pre-treated with 1 nM Baflomycine A1 for 1 hr and incubated with aspirin and/or ABT-737 at the indicated concentrations for 48 hrs. Percentages of apoptotic cells were determined by PI analysis.

**Fig 5:**
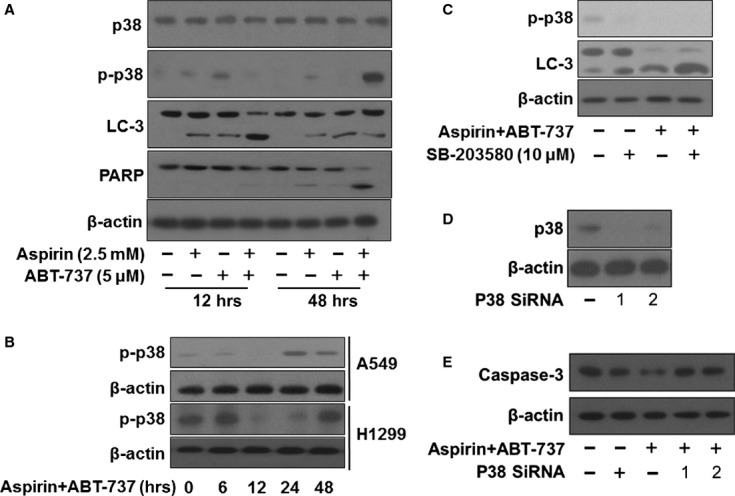
P38 MAPK acted as a switch for the transition from autophagy to apoptosis during combination treatment with aspirin and ABT-737. (A) A549 cells were exposed to aspirin (2.5 mM), ABT-737 (5 μM) or the combination for 12 and 48 hrs, after which protein extracts were immunoblotted using p38, p-p38, LC-3, PARP and β-actin. (B) A549 cells were exposed to aspirin (2.5 mM), ABT-737 (5 μM) or the combination for 6, 12, 24 and 48 hrs, H1299 cells were exposed to aspirin (5 mM), ABT-737 (20 μM) or the combination for 6, 12, 24 and 48 hrs, after which protein extracts were immunoblotted using p-p38 and β-actin. (C) A549 cells were pre-treated with p38 MAPK inhibitor SB-203580 (10 μM) for 1 hr and then treated with 2.5 mM aspirin and/or 5 μM ABT-737 for 12 hrs, after which protein extracts were immunoblotted using p-p38, LC-3 and β-actin. (D) A549 cells were transfected with control SiRNA, p38 siRNA-1 and p38 siRNA-2 according to the manufacturer's recommendations. Forty-eight hours after transfection, cell lysates were prepared for Western blot analysis. (E) The expression of caspase-3 in A549 cells that had been transfected with p38 siRNA and treated with 2.5 mM aspirin, either alone or in combination with 5 μM ABT-737, for 48 hrs were examined.

Several anti-cancer drugs have been shown to induce both apoptosis and autophagy, and autophagy is a mechanism of adaptation to cellular stress and may confer protection from drug-induced cell death 17. We determined whether aspirin and/or ABT-737 could induce autophagy, as detected by expression of the light chain 3 (LC3) protein. We found that aspirin or ABT-737 alone treatment induced conversion of cytoplasmic LC3I to membrane-bound LC3II and the combination of aspirin and ABT-737 induced a greater conversion of LC3I into LC3II than did either drug alone in A549 cells after 12 hrs treatment, but not after 48 hrs treatment, these data indicated that only short-term co-treatment enhanced autophagic response (Figs4A, B and 5A). Consistent with Western blotting results, aspirin plus ABT-737 showed increased percentage of A549 cells displaying punctate GFP-LC3 after 12 hrs relative to either single drug alone (Fig.4C and D). In addition, AO staining was performed to visualize acidic autolysosomes in A549 cells treated with aspirin, ABT-737 or aspirin + ABT-737. Treatment with aspirin and ABT-737 increased autolysosomes both in A549 and H1299 cells as shown by AO staining (Fig.4E and F). Numerous reports implicated the relevance between autophagy and anti-cancer drug resistance 18. Therefore, we determined whether inhibition of autophagy could enhance ABT-737 or aspirin-induced apoptosis. Firstly, we detected whether the autophagy induced by aspirin + ABT-737 could be inhibited by autophagy inhibitors (3-MA and Baflomycine A1). As shown in Figure4G and H, addition of the autophagy inhibitors (3-MA and Baflomycine A1) could effectively inhibit the autophagy induced by aspirin + ABT-737 both in A549 and H1299 cells. Then, A549 and H1299 cells were pre-incubated with 3-MA (1 mM) or Baflomycine A1 (1 nM) for 1 hr and subjected to aspirin, ABT-737 and aspirin + ABT-737, drug-induced apoptosis was determined by PI staining. Autophagy inhibition increased apoptosis induction on either aspirin or ABT-737 single-treatment groups, while 3-MA markedly decreased apoptosis induction by the combination of aspirin plus ABT-737 (Fig.4I). The similar phenomenon was also observed in H1299 cells, indicating that it was not a cell type-specific phenomenon (Fig.4J). Furthermore, Autophagy inhibition by Baflomycine A1 increased apoptosis induction on ABT-737 single-treatment groups, while Baflomycine A1 markedly decreased apoptosis induction by the combination of aspirin plus ABT-737 both in A549 and H1299 cells (Fig.4K and L). This suggested that increased autophagy was correlated with the resistance to aspirin or ABT-737 as single agents, the combination-induced autophagy switched from a cytoprotective signal to a death-promoting signal.

### P38 MAPK acted as a switch for the transition from autophagy to apoptosis during combination treatment with aspirin and ABT-737

To further determine which signalling pathway played an important role in the transition between autophagy and apoptosis with combination treatment of aspirin and ABT-737, we examined several signalling pathways in A549 cells at autophagy (12 hrs treatment) and apoptosis (48 hrs treatment), such as MAPK, PI3K and NF-kB (data not shown). Interestingly, as shown in Figure5A and B, our data revealed that p-p38 was decreased dramatically with 12 hrs of treatment with aspirin + ABT-737 (autophagy time-point), but strongly activated at 48 hrs (apoptosis time-point) both in A549 and H1299 cells. Thus, we hypothesized that p38 kinase might act as a switch in the transition between autophagy and apoptosis in A549 cells treated with aspirin + ABT-737. To further confirm our speculation, A549 cells were pre-treated with p38 MAPK inhibitor SB-203580 (10 μM) for 1 hr and then treated with 2.5 mM aspirin and/or 5 μM ABT-737 for 12 hrs. SB-203580 resulted in an increase in the conversion of LC3I into LC3II with 12 hrs of treatment of aspirin + ABT-737 (Fig.5C), however, p38 siRNA decrease caspase-3 cleavage induced by aspirin + ABT-737 after 48 hrs treatment (Fig.5D and E). Therefore, these data revealed that low p38 activity resulted in aspirin plus ABT-737-induced autophagy and high p38 activity led to aspirin plus ABT-737-induced apoptosis.

### The anti-tumour activity of aspirin and ABT-737 combination therapy against human A549 xenografts

**Fig 6:**
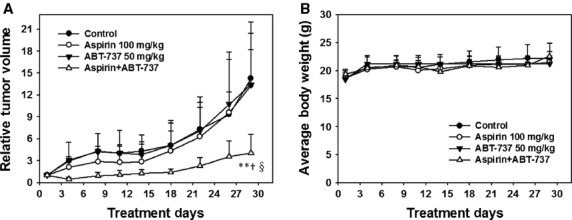
The synergistic effect of aspirin and ABT-737 in a human lung cancer A549 xenograft model. (A) The mice transplanted with A549 human xenografts were randomly divided into four groups and given injection of aspirin (100 mg/kg, i.g.), ABT-737 (50 mg/kg, i.p.), the combination or vehicle for a period of 29 days. Relative tumour volume were expressed as mean ± SD (*n* = 8 per group). **Relative to control, *P* < 0.01; ^†^Relative to aspirin group, *P* < 0.05; ^§^Relative to ABT-737 group, *P* < 0.05. (B) The average bodyweight of each group was expressed as mean ± SD (*n* = 8 per group).

To further characterize the anti-cancer efficacy of aspirin and ABT-737 combination treatment, the *in vivo* activity of aspirin and ABT-737 was detected in a lung cancer A549 xenograft model. As shown in Figure6A, the i.p. administration of ABT-737 at a dose of 50 mg/kg twice per week for 29 days produced no significant difference in mean RTV compared with that of the control group (mean RTV, ABT-737 *versus* control: 13.4 *versus* 14.2; *P* > 0.05). In addition, with the dosage of 100 mg/kg daily for 29 days, aspirin also exerted no significant tumour growth inhibitory effect (mean RTV, aspirin *versus* control: 13.3 *versus* 14.2; *P* > 0.05). As expected, aspirin plus ABT-737 exhibited distinct tumour growth inhibition (mean RTV of combination group *versus* mean RTV of control group: 4.1 *versus* 14.2, *P* < 0.01), significantly greater than aspirin (mean RTV of combination group *versus* mean RTV of aspirin group: 4.1 *versus* 13.3, *P* < 0.05) or ABT-737 treatment (mean RTV of combination group *versus* mean RTV of ABT-737-group: 4.1 *versus* 13.4, *P* < 0.05) alone. Furthermore, compared with the initial bodyweights, combination-treated mice showed no significant bodyweight loss on day 29 (Fig.6B). Thus, the synergistic effect of aspirin and ABT-737 was further validated *in vivo* on A549 xenografts.

## Discussion

Aspirin and other NSAIDs inhibit various tumours growth, but large clinical trials have shown no effect in patients with wild-type PIK3CA cancer 6,19. Thus, for therapy to be effective, approaches to circumvent tumour cell resistance to aspirin in wild-type PIK3CA cancer, such as sensitization by other agents, is needed 20. ABT-737 binds Bcl-2 and Bcl-xL with high affinity and has shown single agent and combination therapy efficacy against multiple myeloma, acute myeloid leukaemia, lymphoma and solid tumour cell lines 21. Here, as shown in Figure1, we demonstrated the combination of aspirin and ABT-737 showed synergistic anti-cancer effects on both PIK3CA wild-type cell lines (A549, H1299, HT-29 and HO-8910) and PIK3CA mutation cell line (HCT-116) *in vitro*. The strong synergistic effect was also validated in a human lung cancer A549 xenograft model. As single agents, aspirin and ABT-737 merely displayed insignificant activities against A549 xenografts, respectively; in contrast, the coadministration of aspirin and ABT-737 exhibited distinct tumour growth inhibition (Fig.6A). Moreover, the combination of aspirin and ABT-737 remarkably improved the anti-tumour capacities *in vivo* without increasing toxicities, as indicated by the nearly constant bodyweights in combination-treated group on day 29 (Fig.6B).

To investigate the mechanism of cytotoxic synergism of combination therapy in PIK3CA wild-type cell lines, we first detected apoptosis by PI staining in the A549 and H1299 cell lines that showed strong synergistic effects with the combination therapy in the cytotoxicity assay. Our results showed that the combination cytotoxicity of aspirin plus ABT-737 in the A549 and H1299 cell lines occurred *via* apoptosis after 48 hrs treatment (Fig.2B and C). In addition, loss of mitochondrial membrane potential was significantly greater with aspirin plus ABT-737 than with either drug used alone (Fig.2D and E). These data indicated that the aspirin + ABT-737 synergy was through mitochondria-mediated apoptosis pathway (Fig.2). Furthermore, combination treatment markedly induced the activation of caspase-3, the cleavage of PARP, the reduction in XIAP both in A549 and H1299 cells (Fig.3). Recent finding showed that aspirin could enhance ABT-263-mediated anti-cancer activity *via* suppression of Mcl-1 in hepatocellular carcinoma 14. Our data showed that the inhibition of Mcl-1 by aspirin + ABT-737 might differ depending on the cell type.

Autophagy is an evolutionarily conserved catabolic pathway that degrades long-lived cellular macromolecules and organelles 22. Under certain stresses, autophagy serves as an alternative cell death mechanism named type II programmed cell death, whereas mounting evidence has shown that autophagy can also act as a cell survival or cytoprotective mechanism, by which cells adapt their metabolism to the stresses induced by starvation, chemotherapeutic agents, or radiation, thus allowing cells to evade apoptosis 23,24. Recent report shows the correlation between autophagy and the resistance of cancer cells to aspirin-induced apoptosis, aspirin induces protective autophagy, a feature of mTOR inhibition 25. Our data suggested that ABT-737 might disrupt aspirin-induced protective autophagy and augment the anti-cancer activity of aspirin. Our data showed that short-term aspirin plus ABT-737 treatment induced a greater autophagic response than did either drug alone in A549 cells (Fig.4). The observation led us to wonder why autophagy increased in aspirin + ABT-737-treated cancer cells. Consistent with previous reports, autophagy inhibition could potentiate the apoptosis induced by ABT-737 or aspirin when cancer cells were resisted to ABT-737 or aspirin by inducing protective autophagy as a self-defence mechanism, while autophagy inhibition markedly decreased apoptosis induction by the combination of aspirin plus ABT-737 in PIK3CA wild-type cell lines A549 and H1299 cells 25,26. The similar phenomenon was also observed in PIK3CA mutation cell line HCT-116 (Fig. S1). This suggested that increased autophagy might be correlated with the resistance to aspirin or ABT-737 as single agents and the combination-induced autophagy could switch from a cytoprotective signal to a death-promoting signal (Fig.4). The molecular switch between cytoprotective autophagy and death-promoting autophagy opened by aspirin and ABT-737 treatment might need further investigation.

The cross-talk between apoptosis and autophagy is quite complex and sometimes contradictory, but surely critical to the overall fate of the cell 9. Key proteins originally thought to be ‘autophagy-related proteins’ are now found to be involved in either inducing or inhibiting apoptosis 27. Similarly, apoptosis inhibiting proteins can also block autophagy-associated cell death 27. P38 can act as a switch between autophagy and apoptosis during chemotherapy 12. As shown in Figure5, we found that aspirin plus ABT-737 induced A549 cells autophagy at an early period (12 hrs), which changed into apoptosis at a later period (48 hrs). Importantly, low p38 activity resulted in aspirin plus ABT-737-induced autophagy and high p38 activity led to aspirin plus ABT-737-induced apoptosis, indicating that p38 acted as a switch for the transition from autophagy to apoptosis associated with aspirin plus ABT-737 treatment time (Fig.5).

In conclusion, we presented evidence showing better therapeutic activity of aspirin when combined with ABT-737 both *in vitro and in vivo*. Therefore, a combination chemotherapy regimen incorporating aspirin with ABT-737 warrants clinical investigation in solid tumours, and may potentially be an efficient therapy for aspirin-resistant PIK3CA wild-type tumours.

## Supporting Information

**Figure S1** (A) HCT-116 cells were pre-treated with 1 mM 3-MA for 1 hr and incubated with aspirin (3 mM) and ABT-737 (3 μM) for 12 hrs, after which protein extracts were immunoblotted using LC-3 and β-actin.
