# Supplementary figures and images for: Role of p38 MAPK in enhanced human cancer cells killing by the combination of aspirin and ABT-737
Source: J Cell Mol Med. 2014 Nov 11;19(2):408–17. doi: 10.1111/jcmm.12461 (PMC4407609; doi:10.1111/jcmm.12461)

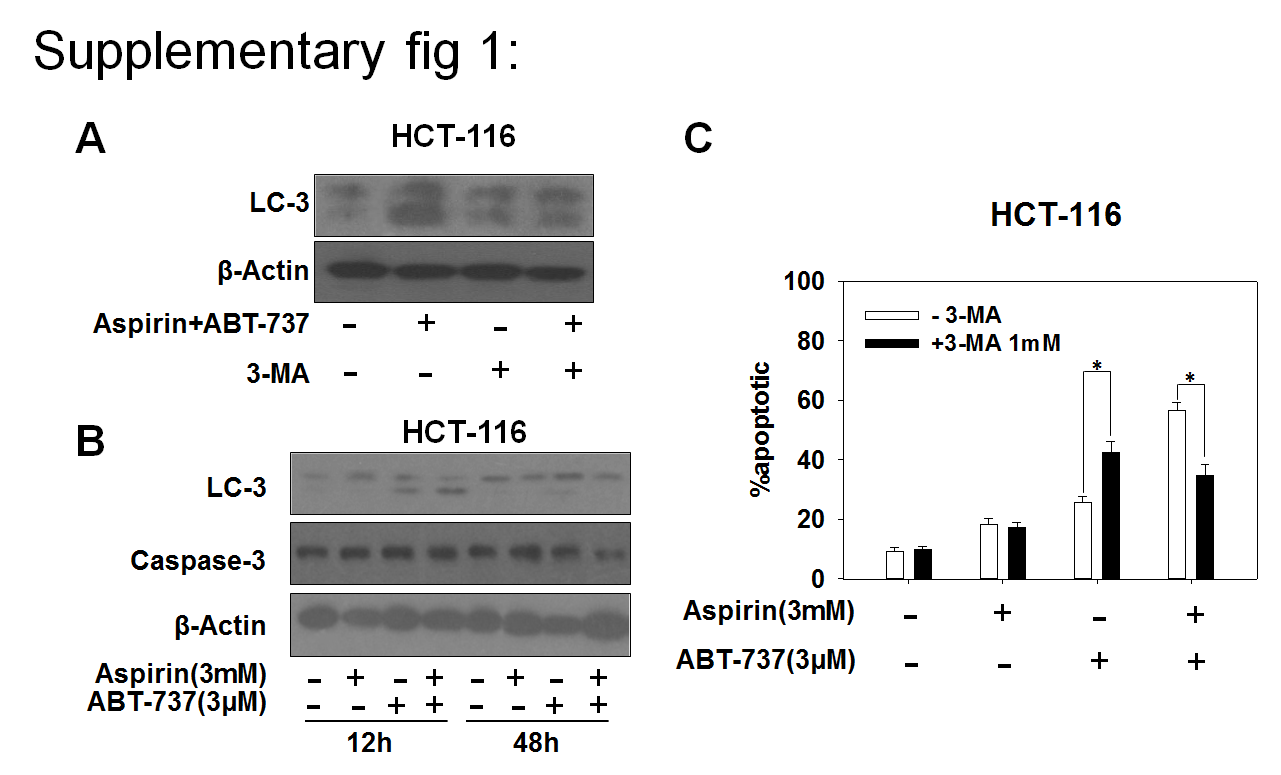

Supplement: Supplementary file 1 [file jcmm0019-0408-sd1.tif]
