# Supplementary material for: Role of p38 MAPK in enhanced human cancer cells killing by the combination of aspirin and ABT-737
Source: J Cell Mol Med. 2014 Nov 11;19(2):408–17. doi: 10.1111/jcmm.12461 (PMC4407609; doi:10.1111/jcmm.12461)
Supplement: Supplementary file 2 [file jcmm0019-0408-sd2.doc]

**Supplementary figure Legends**

**Supplementary figure 1:** (A) HCT-116 cells were pre-treated with 1 mM 3-MA for 1 h and incubated with aspirin (3 mM) and ABT-737 (3 μM) for 12 h, after which protein extracts were immuneblotted using LC-3 and β-actin. (B) HCT-116 cells were exposed to aspirin (3 mM), ABT-737 (3 μM) or the combination for 12 h and 48 h, after which protein extracts were immunoblotted using LC-3, caspase-3 and β-actin. (C) HCT-116 cells were pre-treated with 1 mM 3-MA for 1 h and incubated with aspirin and/or ABT-737 at the indicated concentrations for 48 h. Percentages of apoptotic cells were determined by PI analysis.
